# Magnetic-field-induced phase separation via spinodal decomposition in epitaxial manganese ferrite thin films

**DOI:** 10.1080/14686996.2018.1482520

**Published:** 2018-07-11

**Authors:** Nipa Debnath, Takahiko Kawaguchi, Harinarayan Das, Shogo Suzuki, Wataru Kumasaka, Naonori Sakamoto, Kazuo Shinozaki, Hisao Suzuki, Naoki Wakiya

**Affiliations:** a Graduate School of Science and Technology, Shizuoka University, Hamamatsu, Japan; b Department of Physics, Jagannath University, Dhaka, Bangladesh; c Department of Electronics and Materials Science, Shizuoka University, Hamamatsu, Japan; d Materials Science Division, Atomic Energy Centre, Dhaka, Bangladesh; e School of Materials and Chemical Technology, Tokyo Institute of Technology, Tokyo, Japan; f Research Institute of Electronics, Shizuoka University, Hamamatsu, Japan

**Keywords:** Spinodal decomposition, manganese ferrite thin films, Dynamic Aurora PLD, columnar-type phase separation, *in situ* magnetic field, 40 Optical, magnetic, and electronic device materials, 203 Magnetics / Spintronics / Superconductors, 306 Thin film / Coatings, 504 X-ray / Neutron diffraction and scattering

## Abstract

In this study, we report about the occurrence of phase separation through spinodal decomposition (SD) in spinel manganese ferrite (Mn ferrite) thin films grown by Dynamic Aurora pulsed laser deposition. The driving force behind this SD in Mn ferrite films is considered to be an ion-impingement-enhanced diffusion that is induced by the application of magnetic field during film growth. The phase separation to Mn-rich and Fe-rich phases in Mn ferrite films is confirmed from the Bragg’s peak splitting and the appearance of the patterned checkerboard-like domain in the surface. In the cross-sectional microstructure analysis, the distribution of Mn and Fe-signals alternately changes along the lateral (x and y) directions, while it is almost homogeneous in the z-direction. The result suggests that columnar-type phase separation occurs by the up-hill diffusion only along the in-plane directions. The propagation of a quasi-sinusoidal compositional wave in the lateral directions is confirmed from spatially resolved chemical composition analysis, which strongly demonstrates the occurrence of phase separation via SD. It is also found that the composition of Mn-rich and Fe-rich phases in phase-separated Mn ferrite thin films deposited at higher growth temperature and *in situ* magnetic field does not depend on the corresponding average film composition.

## Graphical abstract

**Figure:**
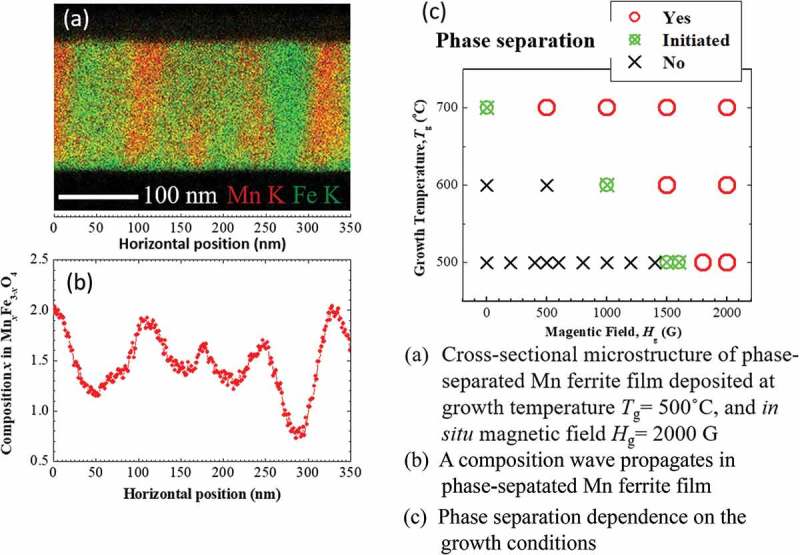

## 1. Introduction

Fabrication of self-assembled periodic microstructure in material systems has attracted much attention in recent years due to its potential applications in the development of functional materials and nanodevices. Mechanical, electrical, and magnetic properties of ceramics depend not only on the properties of the constituent phases but also on the microscopic structure. Hence, to improve the functionalities of ceramic-based devices, it is necessary to control their structure at nanoscale level. Spinodal decomposition (SD) was first explored theoretically and experimentally by Cahn [1–5]. SD is a phase separation process whereby a material system spontaneously separates into two phases with distinct compositions through a non-equilibrium process which can be exploited to control the microstructure at the nanometer scale. Unlike conventional phase separation, which occurs via nucleation and growth, SD does not require nucleation and is only determined by up-hill diffusion with compositional fluctuations. The modulation of composition leading to the spontaneous formation of the periodic microstructure is the characteristic behavior of spinodally decomposed compounds or alloys. It is fascinating that the spontaneously formed periodic structure influences the band gap, transport, and optical properties of the semiconductor alloys [6]. Additionally, periodically formed ferromagnetic alloys after SD wherein a magnetically ordered phase is embedded in a non-magnetic matrix is a very promising structure for device applications because giant magnetoresistance and other collective properties were observed, and thus has important implications for device design [6–8]. SD is a general phenomenon observed in various systems, such as alloys, glasses, and oxides. It is well-established theoretically and experimentally that SD could occur generally in material systems whose phase diagram shows a miscibility gap, such as Al_2_O_3_-Cr_2_O_3_ [9], TiO_2_-SnO_2_ [10], HfO_2_-SiO_2_ [11], and CoFe_2_O_4_-Co_3_O_4_ [12,13].

Of particular intense interest is to create periodically structured materials at the nanometer scale through spontaneous processes during thin film growth. There is an interesting example of SD where phase diagram does not indicate the miscibility gap in bulk Ga_1-*x*_Al*_x_*As system, but Ga_1-*x*_Al*_x_*As semiconductor alloy undergoes phase separation via SD during thin film growth [14]. Therefore, the fabrication of periodic nanostructure by phase separation through SD during thin film growth in such material systems whose phase diagram of the bulk system has no spinodal region is very intriguing, and this research opens new, still largely unexplored ways for creating new materials with potentially important properties.

Generally, SD can be induced or promoted in material systems through prolonged post-deposition annealing process. For example, the preparation of spinodally decomposed thin films of spinel-structured cobalt ferrite (Co ferrite) thin films by long-time post-deposition annealing has already been established [12,13]. However, several-steps synthesis procedures are detrimental to the fabrication of these materials in large scale for commercial applications. Many researchers have paid more attention to self-organized one-step fabrication techniques to fabricate spinodally decomposed compounds or alloys. SD can be controlled or promoted by ion-impingement [15] or ion-bombardment [16] during thin film growth. On the other hand, we have developed a novel pulsed laser deposition (PLD) system by introducing an electromagnet in a PLD vacuum chamber. We can apply the magnetic field during thin film growth in this sophisticated PLD system which is named as Dynamic Aurora PLD [17–21]. In Dynamic Aurora PLD, ion-impingement to the film can be enhanced through suppression of the recombination of ions and electrons in the plume by application of *in situ* magnetic field during deposition. We have reported about the spontaneous superlattice formation in Sr_1+*y*_TiO_3+*y*_ films deposited under *in situ* magnetic field using Dynamic Aurora PLD, which can be explained by SD along the out-of-plane direction [17]. We have also reported that SD can be enhanced in Co ferrite thin films by applying *in situ* magnetic field during the film growth without post-deposition annealing [20]. Therefore, it might be expected that application of *in situ* magnetic field during film growth can induce phase separation in case of other spinel ferrites. We focused on manganese ferrite (Mn ferrite) for the present study because MnFe_2_O_4_-Mn_3_O_4_ system has a wide composition range in spinel structure similar to CoFe_2_O_4_-Co_3_O_4,_ though there are no reports about phase separation via SD in bulk Mn ferrite [22]. The purpose of this work is to clarify the occurrence of spontaneous phase separation via SD in Mn ferrite films grown under *in situ* magnetic field by Dynamic Aurora PLD.

## 2. Experimental details

Mn*_x_*Fe_3-*x*_O_4_ thin films were deposited on MgO (001) substrates under a fixed *in situ* magnetic field of 0– 2000 G. The reason why we chose MgO (001) as a substrate is that twice of the lattice parameter of MgO (0.4217 nm, ICCD card no: 01-071-1176) nearly match with that of the bulk Mn ferrite (0.08515 nm, JCPDS file no: 73–1964) which results in a small lattice mismatch of 0.81%. The design and details of Dynamic Aurora PLD have been described in some of our previous reports [17–21]. The direction of the *in situ* magnetic field is parallel to the direction from the target to the substrate. Mn ferrite ceramic targets were prepared using a conventional solid-state reaction. The target was irradiated with a focused Nd:YAG pulsed laser (λ = 266 nm). The laser fluence was approximately 2 J/cm^2^. The repetition rate was 10 Hz. The oxygen gas pressure in the vacuum chamber was controlled at $1\times 10^{-4}$ Torr during deposition. The growth temperature (*T*_g_) was 500–700 °C. The deposition time was 20–120 min. The deposition time was varied to adjust the film thickness. The thickness of the Mn ferrite films was between 80 and 200 nm. The crystal structure of the thin films was examined via reciprocal space mapping (RSM) that was performed with a high-resolution X-ray diffractometer (XRD: ATX-G; Rigaku Corp., Japan). An X-ray fluorescence spectrometer (XRF: Minipal; PANalytical B.V., The Netherlands) was used to estimate the composition and thickness of all as-grown films. The surface morphology of Mn ferrite thin films was examined by an atomic force microscope (AFM: SPA400; Hitachi Ltd., Japan). The cross-sectional morphology and corresponding elemental composition of Mn ferrite films were studied using a scanning transmission electron microscope (STEM: JEM-2100F; JEOL Ltd., Japan) equipped with an energy dispersive spectroscopy (EDS) detector. The microscope was operated at an accelerating voltage of 200 kV. The samples for STEM analysis were prepared using a focused ion beam (FIB: JIB-4500; JEOL Ltd., Japan) system. Before employing FIB technique, a thin layer of osmium (Os) was coated on the surface of the samples to protect them from damage during FIB milling and to increase their conductivity. The composition analysis of the samples was performed by the line-scan analysis of STEM-EDS maps. The spatial resolution of EDS composition analysis was approximately 6 nm (Figure S1 in supplementary information).

## 3. Results and discussion

### 3.1 Phase and structural analyses

**Figure 1.**
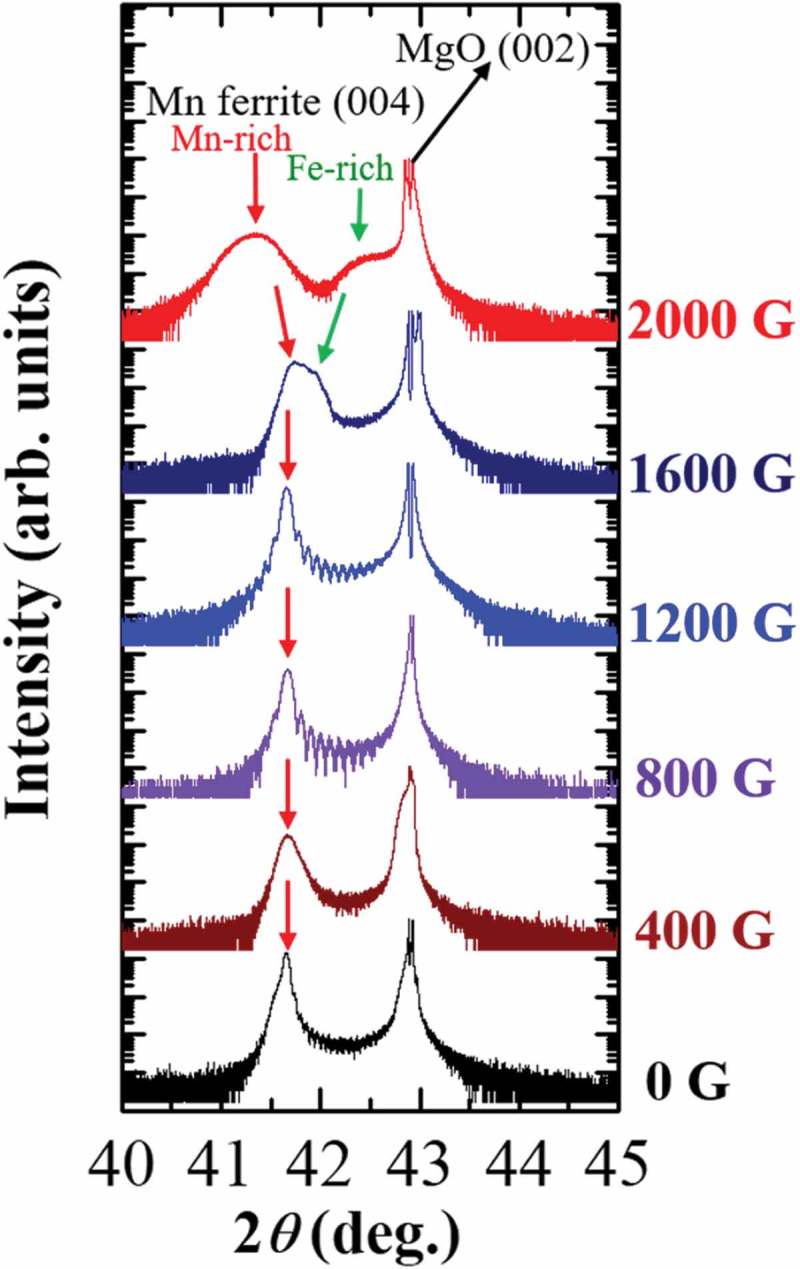
*θ*-2*θ* XRD patterns of Mn ferrite thin films with *x* = 1.45 deposited under different strength of magnetic field on MgO (001) substrate at 500 °C growth temperature.

Figure 1 presents the XRD patterns of Mn ferrite thin films with *x* = 1.45 grown on MgO (001) substrate at *T*_g_ = 500 °C and different strength of magnetic field. For convenience, the films are labeled according to the value of magnetic field applied during their growth. All as-grown Mn ferrite thin films were deposited along the *c*-axis which is demonstrated by the presence of the strong Mn ferrite (004) peaks. At *in situ* magnetic field lower than 1200 G, Laue fringes beside the (004) peak are observed, whose oscillation period is consistent with the film thickness. Thus, these thin films comprise single phases of Mn ferrite. On the other hand, the Bragg’s peak (004) of 1600 G film becomes broader and starts to split, and the (004) peak of 2000 G Mn ferrite film is completely divided into two peaks. The peak splitting indicates that the phase separation occurs during the Mn ferrite thin film growth, similar to our previous report on Co ferrite films grown using Dynamic Aurora PLD [20]. Since the peak broadening typically arises from the non-uniform strain, here, the continuous gradual change of interplanar spacing *d*, which arises due to a continuous composition variation in samples, is a possible reason that Mn-rich or Fe-rich phase-indicting peak becomes broader after phase separation. It is known that the lattice parameter (*c*-axis) increases with increasing Mn-content in Mn*_x_*Fe_3-*x*_O_4_ [23,24]. Conversely, the increase of Fe-content in Fe*_x_*Mn_3-*x*_O_4_ reduces the lattice parameter [25]. The increase of lattice parameter (*c*-axis) means the shifting of Bragg peaks toward the lower 2*θ* angle and vice versa. From the XRD patterns of 1600 and 2000 G films in Figure 1, the peak position of lower 2*θ* angle side (assuming one phase) of 1600 G film is shifted to lower 2*θ* angles in 2000 G film. On the other hand, another peak of higher 2*θ* angle (assuming another phase) of 1600 G film is shifted to larger 2*θ* angles in 2000 G Mn ferrite film. The red and green arrows in Figure 1 are used to indicate this scenario. From the above discussion, it can be assumed that Mn ferrite (004) peaks at lower and higher 2*θ* angles side in the XRD pattern of 2000 G Mn ferrite film correspond to Mn-rich and Fe-rich phases, respectively. Moreover, it can be observed from Figure 1 that the MgO (002) peak seems to split or shrink in some XRD patterns. This can be explained by two reasons. It has been mentioned in experimental details that XRD analysis was performed by ATX-G where the movement of automatic attenuator sometimes failed, resulting in an apparent splitting of the MgO peak. In addition, since the intensity of MgO single-crystal substrate is very high and our ATX-XRD system has the limitations of detecting this high intensity, consequently shrinking of MgO peak occurs. On the other hand, face-centered-cubic structure MgO single-crystal substrate could possess mosaicity, which might induce a splitting in the MgO peak.

**Figure 2.**
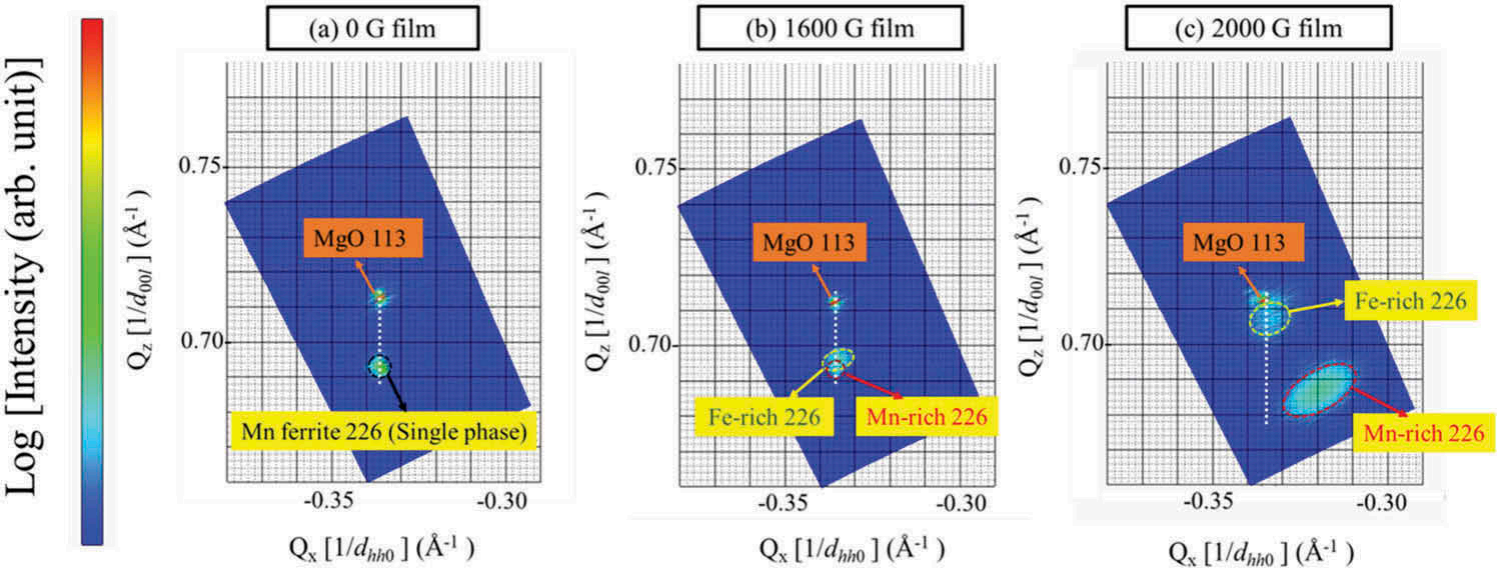
RSM images of (a) 0 G, (b) 1600 G, and (c) 2000 G Mn ferrite thin films around Mn ferrite 226 and MgO 113 diffraction conditions. The white dashed lines indicate in-plane matching to the substrate. Reciprocal spots for Mn ferrite films are marked by circular dashed lines which are determined by *Q*_z_ values obtained from corresponding *d*-spacings of the XRD patterns.

RSM images of Mn ferrite films around the MgO (113) and Mn ferrite (226) planes for 0, 1600, and 2000 G Mn ferrite films are depicted in Figure 2, where the horizontal and vertical axes correspond to the reciprocal of the in-plane and out-of-plane lattice spacings, respectively. The presence of Mn ferrite 226 reflexes in these RSM images demonstrates that all Mn ferrite films are epitaxially grown on the MgO (001) substrate. As shown in Figure 2(a), the position of the film peak along the horizontal axis, i.e. *Q*_x_ value is the same as that of the substrate peak, which means that the 0 G Mn ferrite film was grown coherently on MgO, having the same in-plane lattice constant. As seen in Figure 1, 1600 G Mn ferrite film shows the peak splitting, but this is only initiated and thus two reciprocal points in Figure 2(b) are not clearly distinguished. The peak positions of two phases in the RSM image have been determined by *Q*_z_ values calculated from the corresponding *d*-spacings from the peak positions of Fe-rich and Mn-rich phases in the XRD pattern of 1600 G Mn ferrite film. It can be seen that both phases and MgO substrate are found to possess the same *Q*_x_ values for their diffraction peaks, which is also indicative of in-plane matching alignment to the substrate. On the other hand, two different reciprocal spots are identified in the RSM image of 2000 G Mn ferrite film (Figure 2(c)) which confirm the presence of two distinct phases of different lattice parameter. It should be noted here that Fe-rich phase is matched to MgO 113 diffraction spot having same *Q*_x_ value (−0.335), while Mn-rich phase has different *Q*_x_ value (−0.320) from the substrate. The reciprocal spot of Mn-rich phase is shifted to smaller |*Q*_x_| values which correspond to almost same lattice parameter as the out-of-plane one, indicating the lattice of the Mn-rich phase is almost relaxed. It is noteworthy to state that the lattice of Mn-rich phase expands and deviates from the MgO lattice parameter with increasing the Mn content due to the phase separation, leading to a larger lattice mismatch (2.77%) with MgO substrate. We reported similar phenomena in Co ferrites, where the Fe-rich phase in a 2000 G film was relaxed though a 0 G film with single phase showed in-plane matching with MgO substrate [20].

### 3.2 Surface and cross-sectional microstructure analyses

**Figure 3.**
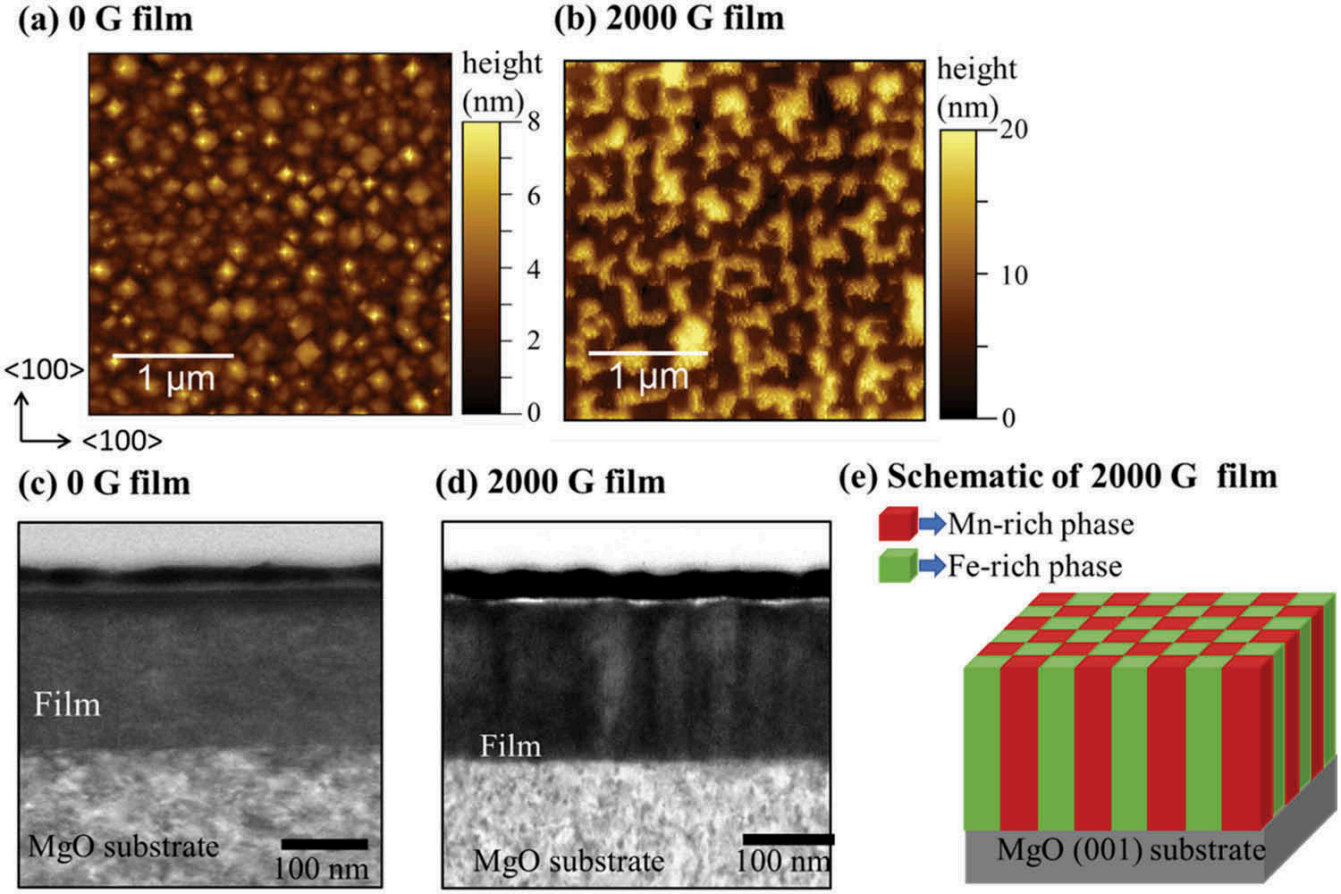
AFM surface morphology of (a) 0 G, (b) 2000 G Mn ferrite thin films, and bright-field (BF) cross-sectional TEM images of (c) 0 G, (d) 2000 G Mn ferrite films, and (e) a schematic of the phase-separated 2000 G Mn ferrite film.

Microstructure analysis of Mn ferrite films was performed using AFM and STEM. The surface morphologies of 0 and 2000 G films are presented in Figure 3(a, b). The 0 G Mn ferrite film has a pyramid-like domain structure which seems to be faceted along (111) planes. Previously, it was reported that spinel oxide nanostructures of CoCr_2_O_4_ on MgO (001) [26] or CoFe_2_O_4_ on SrTiO_3_ (001) [27] substrates tend to grow into pyramid-like grains along {111} planes to minimize the surface energy. It has to be noted that the surface energy in spinels is strongly anisotropic, with the {111} planes having the minimal surface energy [27]. The root-mean-square (rms) roughness is 1.0 nm for 0 G film. On the other hand, 2000 G Mn ferrite film has a checkerboard-like domain in surface structure. The patterned surface structure is the evidence of composition modulation, which demonstrates the spinodal nature of the film grown under magnetic field [1–5]. The AFM image of 2000 G phase-separated film demonstrates the patterned surface morphology where Mn-rich phase and Fe-rich phase are arranged in a periodic manner. The rms roughness of 2000 G film is 5.0 nm, which is higher than that of 0 G Mn ferrite film. Presumably, it can be said that the rms roughness increases due to the transition of single-phase structure of 0 G film to double-phase structure of 2000 G Mn ferrite film wherein the Mn-rich and Fe-rich phases may have the different height. The two-phase structure of Mn ferrite film with different height has a significant contribution to the overall surface roughness. The bright-field cross-sectional TEM images for both films are shown in Figure 3(c, d). The 0 G film has homogeneous contrast in the whole film region as seen in Figure 3(c). In the cross-sectional image of 2000 G Mn ferrite film (Figure 3(d)), darker and brighter vertical regions are alternately ordered, implying columnar-type phase separation. Based on the above microstructural analysis, we have drawn a schematic of phase-separated Mn ferrite film in Figure 3(e) to illustrate the periodic microstructure after phase separation.

**Figure 4.**
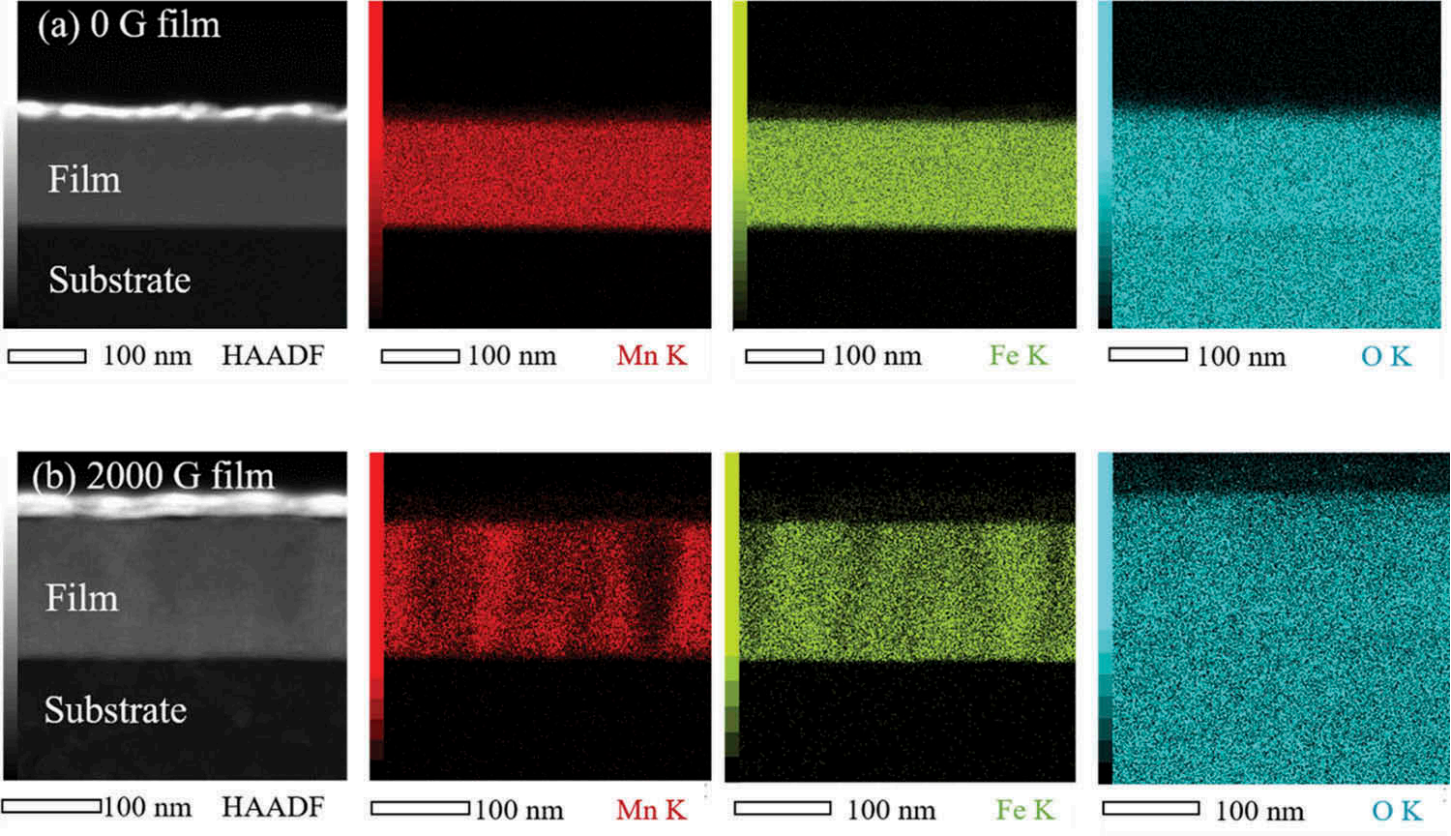
Cross-sectional STEM image and corresponding EDS elemental maps of Mn K, Fe K, and O K signals for (a) 0 G and (b) 2000 G Mn ferrite films, where Mn, Fe, and O elements are represented as red, green, and cyan colors, respectively.

To confirm the columnar-type phase separation, STEM-EDS elemental analysis was conducted. Figure 4 displays the cross-sectional high angle annular dark field (HAADF) STEM images of the Mn ferrite films and corresponding EDS maps of Mn, Fe, and O elements. In HAADF images of both 0 and 2000 G Mn ferrite films, the top white color layer corresponds to Os. The intensity of O element is evenly distributed throughout the entire Mn regions in both 0 and 2000 G films. In addition, the distribution of Fe K and Mn K intensity in 0 G Mn ferrite film is flat and homogeneous. On the other hand, Mn and Fe maps of 2000 G film (Figure 4(b)) reveal stripe-like patterns where Mn-rich regions correspond to Fe-poor areas. This observation confirms the columnar phase separation in 2000 Mn ferrite film.

**Figure 5.**
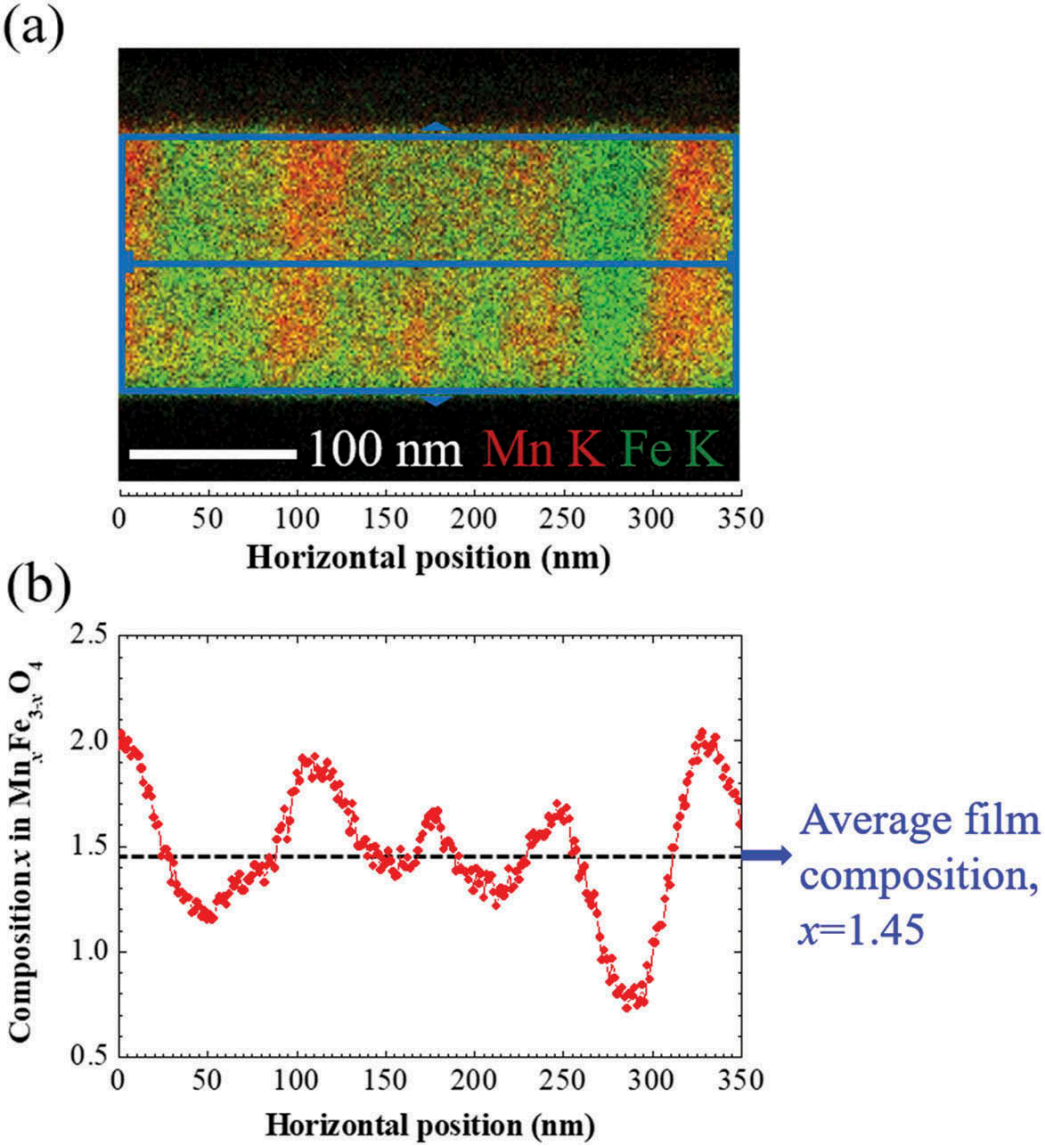
(a) Superimposed STEM-EDS maps of Mn K and Fe K in the 2000 G film shown in Fig. 4(b). (b) Line-profile of composition *x* in Mn*_x_*Fe_3-*x*_O_4_ of 2000 G Mn ferrite film as a function of horizontal position in EDS maps of (a). The composition at each horizontal position is obtained using both Mn K and Fe K intensities which are averaged along the vertical direction. The black dashed line indicates the average composition of 2000 G Mn ferrite film determined from XRF measurement.

To determine the composition of each phase formed after phase separation, we used the EDS elemental spectra obtained from the STEM-EDS analysis. Figure 5 displays the variation of composition *x* along a horizontal direction in Mn*_x_*Fe_3-*x*_O_4_ of 2000 G Mn ferrite thin film. The composition of each point at horizontal position in Figure 5(a) is averaged along the vertical direction because the composition modulation occurs almost only in the lateral directions in 2000 G film which can be mentioned from Figure 4. The composition of each phase does not abruptly change but fluctuates up and downward continuously from the average film composition 1.45 which is determined using the XRF measurement as mentioned above. The composition wave propagates with the oscillation period of about 100 nm along horizontal direction. Here, compositional non-homogeneity occurs because of compositional wave propagation. Figure 5(b) reveals the most crucial evidential fact that the composition wave propagates sinusoidally along the in-plane (lateral) directions (x- and y-directions), which is the characteristic feature of SD [1–5]. In this present situation of phase-separated Mn ferrite film, i.e. the progression of the sinusoidal composition profile during SD is contrast to the sequences of the formation of two phases by nucleation and growth in where the phase separation arises from a discontinuous composition variation. It is worthwhile to mention that the composition *x* of Mn-rich phase approaches to about 2 and that of Fe-rich to 1 or less than 1, which is close to the compositions of FeMn_2_O_4_ and MnFe_2_O_4_, respectively.

From the above analysis of the microstructure of phase-separated Mn ferrite film, it can be clearly concluded that the columnar phase separation occurs in Mn ferrite film and SD is the mechanism of this phase separation. The reason why the SD wave propagates almost exclusively along lateral directions is still not clear, but we can mention some reports related to this phenomenon. He et al. have demonstrated that different nanoscale compositional patterns can be formed in the film after phase separation and such nanoscale patterning is attributed to the competition of phase separation and surface segregation, as well as to the geometrical confinement of the diffusion in the film growth [28]. When the diffusion along the surface is dominant, the film tends to form cylinder or columnar structures perpendicular to the growth plane as diffusion is confined in the top layers, whereas the subsurface interdiffusion (diffusion normal to the surface (z-direction)) is negligible. Lu et al. have developed a phase-field-model of SD in epitaxial films and established the relationship among morphologies of two-phase microstructure, alloy composition, and deposition rate with the aid of computer simulations [29]. According to their simulations results, the lateral composition modulation develops at a slower deposition rate relative to the phase separation process in the films which is also consistent with the simulation results reported in Ref. [28]. The case of the Mn ferrite film growth using Dynamic Aurora PLD would correspond to this typical case and thus columnar-type phase separation can be found. On the other hand, when the growth rate is higher, the composition variation occurs in the direction normal to the film plane. This phenomenon, called surface-directed spinodal decomposition (SDSD), generates the superlattice structure in the film after phase separation [17,29]. In our previous study, we reported the superlattice structure formation through SDSD in SrTiO_3_ films grown by Dynamic Aurora PLD [17]. Here, the composition wave propagates in the vertical direction [17,28,29].

### 3.3 Discussion of the mechanism of phase separation in Mn ferrite system

**Figure 6.**
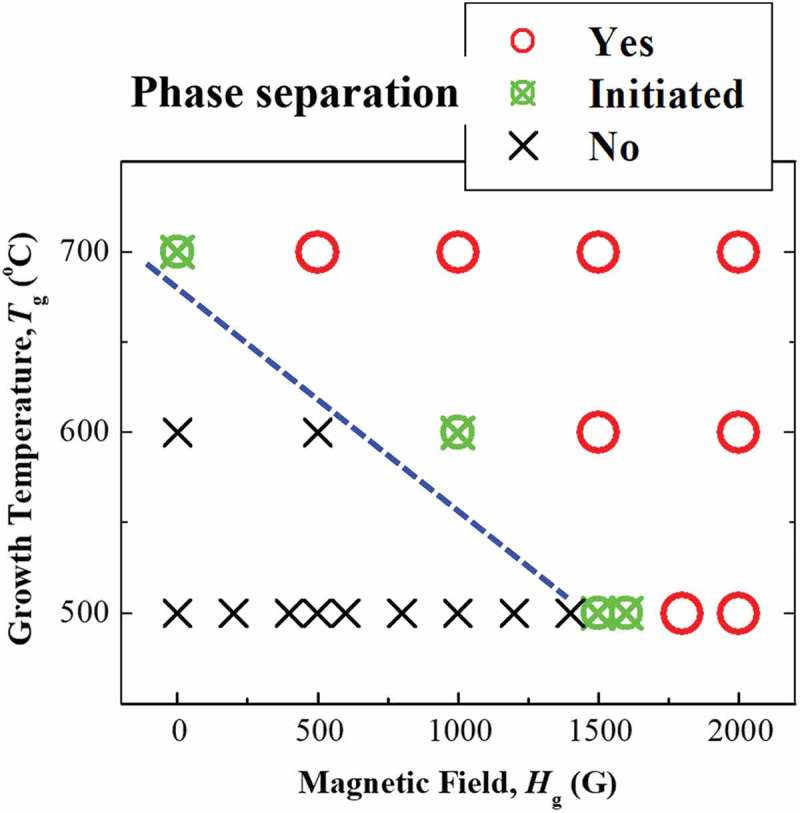
Phase separation in Mn ferrite films as a function of the growth temperature and magnetic field applied during the film growth. Red open circles, green crossed circles, and black crosses indicate that the phase separation was confirmed, initiated, and not observed, respectively. The boundary between the phase-separated and non-separated system is indicated by the blue dashed line.

To confirm the mechanism of the spontaneous phase separation in current Mn ferrite films more precisely, we have summarized that phase separation occurred or not by changing the growth temperature, *T*_g_ and the magnitude of *in situ* magnetic field, *H*_g_ during film growth in Figure 6. The occurrence of phase separation was confirmed from the existence of peak splitting in the XRD patterns of these Mn ferrite thin films. In Figure 6, the term ‘Initiated’ is used to indicate an intermediate stage of phase separation, where the (004) XRD reflection starts splitting, but the split components are not fully separated. For 500 °C, this value of the magnetic field to cause phase separation is 1500 G. At 600 °C, 1000 G magnetic field is enough to initiate phase separation. Thus, Figure 6 indicates that the phase separation occurs at lower magnetic fields with increasing *T*_g_. Chen et al. reported that SD can be induced by ion-impingement during the deposition of amorphous TiC thin films by pulsed DC magnetron sputtering. They also found that the energy flux of impinging ions enhances the diffusion coefficient [15]. They have tentatively expressed ion-impingement-enhanced diffusion coefficient as [15,17]

(1) $$D*=D_{0}exp\left(-\frac{E-\alpha I}{kT_{g}}\right)$$

where *D_0_* refers the pre-exponential factor, *E* is the activation energy, *I* is the energy flux by the ion-impingement, *α* is a positive constant, *k* is Boltzmann constant, and *T*_g_ stands for growth temperature. This equation indicates that ion-impingement during thin film growth lowers the activation energy for up-hill diffusion and SD is consequently induced to bring about spontaneous phase separation [15,17,20]. It should be noted that the application of a magnetic field to the laser-ablated plume suppresses the recombination of electrons and ions. Therefore, ion-impingement occurs during thin film deposition using Dynamic Aurora PLD [17,20]. In addition, an increase in *in situ* magnetic field leads to increase the flux density of impinging ions due to the Lorenz force. In the present case, both reduction in the activation energy and the increase in the growth temperature can enhance the diffusion coefficient, which is consistent with the dependence of phase separation on *T*_g_ and *H*_g_ presented in Figure 6. It is observed that at high *T*_g_ = 700 °C, the phase separation is initiated in the thin film without *in situ* magnetic field, although no SD has been reported in bulk Mn ferrites. This may happen because of a weak ion-impingement, which occurs during PLD even in the absence of magnetic field [20,30]. Negi et al. reported the inhomogeneity of films grown by PLD from a Co:ZnO target. The films comprised three major phases: Co metal, Co oxides, and Co-doped ZnO [30]. However, our results are different with respect to the film inhomogeneity and phase separation.

**Figure 7.**
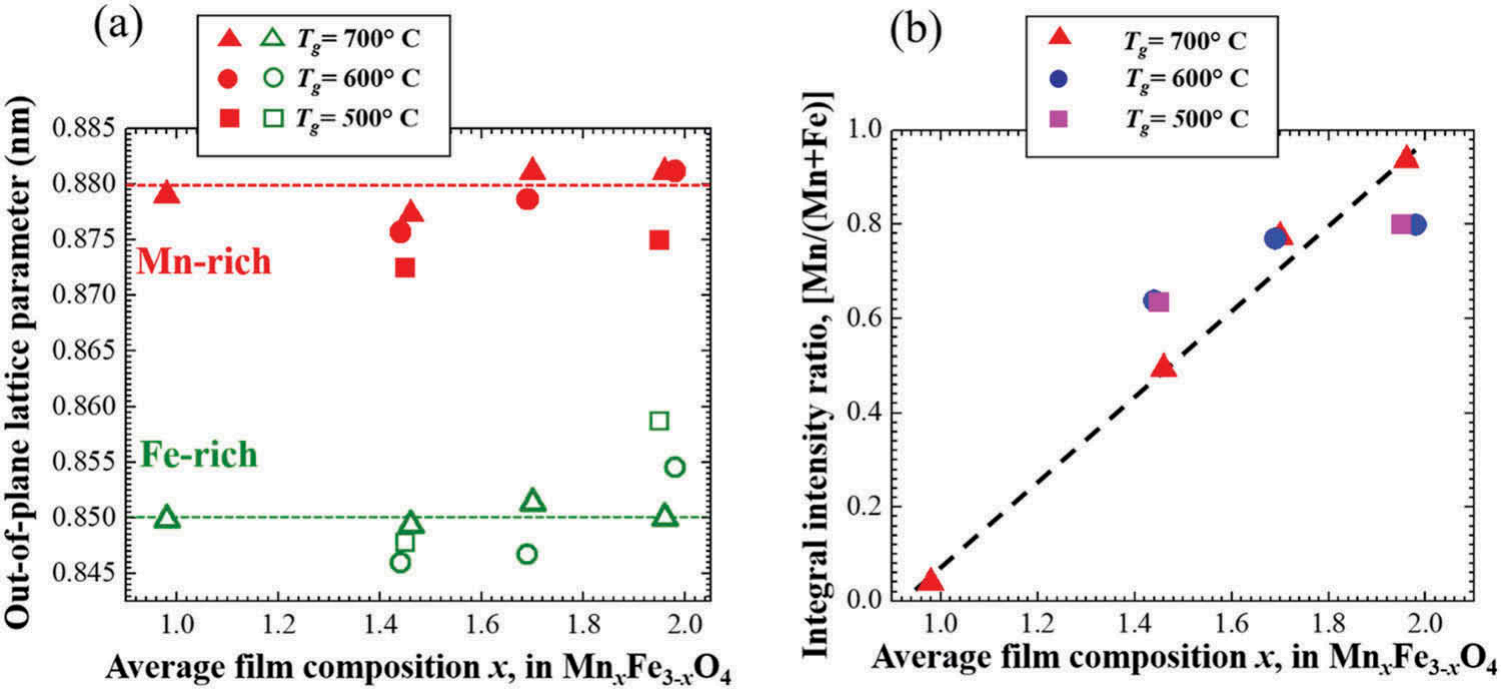
(a) Out-of-plane lattice parameters and (b) integral XRD intensity ratios of Mn-rich and Fe-rich phases in 2000 G Mn ferrite films of different composition deposited at different *T*_g_. In (a), the red and green dashed lines imply the lattice parameter of compositions at Mn-rich and Fe-rich sides on spinodal line in MnFe_2_O_4_-Mn_3_O_4_ system, respectively. In (b), the black dashed line is used to guide the reader’s eyes.

Figure 7(a) shows the out-of-plane lattice parameters of Mn-rich and Fe-rich phases in phase-separated Mn ferrite of different average film composition deposited under 2000 G magnetic field at 500–700 °C growth temperature. The out-of-plane lattice parameters of Mn-rich and Fe-rich phases are calculated from the corresponding 2*θ* values of the Bragg peaks in XRD patterns. We have mentioned in Section 3.1 that the Mn-rich and Fe-rich phases should have larger and smaller lattice parameters, respectively. From Figure 7(a), it is also confirmed that the lattice parameter of Mn-rich phase is larger than that of Fe-rich phase in case of all phase-separated films of different composition. The lattice parameters of Mn-rich phase in all Mn ferrite films deposited at 700 °C are almost same. The 500 °C and 600 °C films have similar or smaller out-of-plane lattice parameters of Mn-rich phase. The lattice parameters of Fe-rich phase in all 700 °C thin films are also independent on average film composition. It is found that the out-of-plane lattice parameters of Fe-rich phase scatter a little at other growth temperatures in all phase-separated Mn ferrite films. Lattice distortions in the Fe-rich phase were confirmed by RSM images, and its in-plane lattice parameters approach those of MgO, leading to a close in-plane matching with the substrate. Hence, it can be claimed that whatever the average film composition is, the out-of-lattice parameters of Mn-rich phase in all phase-separated Mn ferrite films have almost same values indicated by the red dashed line in Figure 7(a). And the similar fact is also true for the lattice parameters for Fe-rich phase in Mn ferrite films indicated by the green dashed line in Figure 7(a). In addition, there is a significant difference between the composition (lattice parameters) of two phases in phase-separated Mn ferrite films. The miscibility gap in the phase diagram of any material system defines the composition range within which a homogeneous solid solution becomes unstable [2]. Presumably, the compositions of Mn-rich and Fe-rich phases in spinodally decomposed Mn ferrite films maintain a certain range. It can be qualitatively said that this range of composition of phase-separated Mn ferrite films can be defined as miscibility gap in MnFe_2_O_4_-Mn_3_O_4_ system.

Figure 7(b) shows average film composition dependence of the integral peak intensity ratio of the (Mn-rich)/(Mn-rich + Fe-rich) phases in *θ*-2*θ* XRD patterns. With increasing Mn content in the film, the ratio increases almost linearly. The ratio roughly corresponds to the volume fraction of Mn-rich phase. It can be said qualitatively that the change of the volume fraction of two phases coincides with the average film composition. Because, as shown in Figure 7(a), the compositions of both Mn-rich and Fe-rich phases are independent on the average film composition, the volume fraction of Mn-rich phase should increase with average film composition. Therefore, the linear increase in the ratio is very reasonable for maintaining the average composition value of the Mn ferrite films. Notably, the ratio tends to be 0 at about *x *= 1 and be 1 at about *x *= 2 in Mn*_x_*Fe_3-__*x*_O_4_ as seen in Figure 7(b). As shown in Figure 5, the composition modulation approaches *x* = 1 and *x* = 2 in alternate Mn- and Fe-rich regions. This observation supports possible implication for the composition range of the miscibility gap of the Mn ferrite system during the phase separation due to magnetic-field-induced ion-impingement.

## 4. Conclusions

Spontaneous phase separation occurs during the growth Mn ferrite films by magnetic-field-induced ion-impingement. The underlying mechanism of this phase separation is the SD (up-hill diffusion) that is confirmed by composition fluctuations, the patterned surface structure of phase-separated Mn ferrite films. Cross-sectional images of phase-separated Mn ferrite films reveal a columnar nanostructure where in alternate columnar-like domains of Mn-rich and Fe-rich phases were vertically aligned on MgO (001) substrate. The composition wave propagates sinusoidally along in-plane directions (x- and y-directions) which is demonstrated from the lateral composition modulation in spinodally decomposed Mn ferrite thin film. Phase separation occurs at lower *in situ* magnetic fields with increasing growth temperature. Thus, the occurrence of phase separation can be explained from the enhancement of diffusion coefficient on the surface by thermally and the magnetic-field-induced ions flux energy. Moreover, we provide a possible scenario that a spinodal line in Mn ferrite films lies near MnFe_2_O_4_ and FeMn_2_O_4_, from the analyses of STEM-EDS line profiles, and the average film composition dependence of the lattice parameters and volume fraction of Mn-rich and Fe-rich phases in phase-separated Mn ferrite films. This work provides an effective pathway to understand and control of phase separation in other ferrites or ceramic thin films.
