# Supplementary material for: Magnetic-field-induced phase separation via spinodal decomposition in epitaxial manganese ferrite thin films
Source: Sci Technol Adv Mater. 2018 Jul 11;19(1):507–16. doi: 10.1080/14686996.2018.1482520 (PMC6041787; doi:10.1080/14686996.2018.1482520)
Supplement: Supplemental Material [file TSTA_A_1482520_SM1013.pdf]

Supplementary Materials

Magnetic-field-induced phase separation via spinodal decomposition in epitaxial manganese ferrite thin films

Nipa Debnath[a,b*], Takahiko Kawaguchi[c], Harinarayan Das[d], Shogo Suzuki[c], Wataru Kumasaka[c], Naonori Sakamoto[c,f], Kazuo Shinozaki[e], Hisao Suzuki[a,c,f], and Naoki Wakiya[a,c,f**]

[a] *Graduate School of Science and Technology, Shizuoka University, Hamamatsu, 432-8561, Japan*

[b] *Department of Physics, Jagannath University, Dhaka. 1100, Bangladesh*

[c] *Department of Electronics and Materials Science, Shizuoka University, Hamamatsu, 432-8561, Japan*

[d] *Materials Science Division, Atomic Energy Centre, Dhaka-1000, Bangladesh*

[e] *School of Materials and Chemical Technology, Tokyo Institute of Technology, Tokyo, 152-8550, Japan*

[f] *Research Institute of Electronics, Shizuoka University, Hamamatsu, 432-8561, Japan*

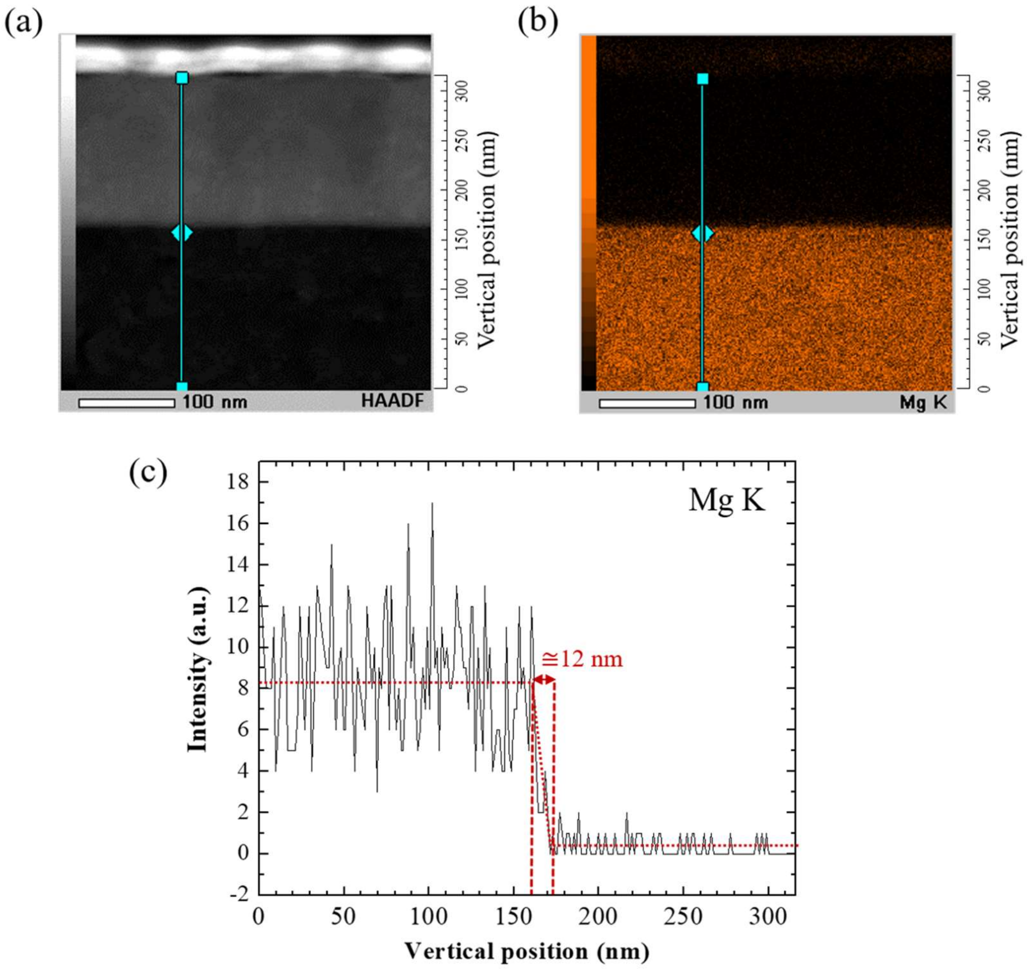

Figure S1. (a) High angle annular dark field (HAADF) STEM image of 2000 Mn ferrite film, (b) corresponding EDS elemental map of Mg K in film, (c) Line-profile as a function of vertical position in EDS maps of Figs. 5(a, b) indicated by cyan colored line.

Figure S1 (a) is the same image shown in Fig. 5(a). Figure S1 (b) is the corresponding EDS map for Mg K of 2000 G Mn ferrite film. Figure S1 (c) is the line profile of Mg K along the direction perpendicular to the substrate. The scanned line is shown in Figs. S1(a, b) as a cyan colored line. Though the intensity of Mg K is rather noisy, but steep-drop is observed at the film/substrate interface. From this data, we have estimated the spatial resolution of STEM-EDS which is around 12 nm. As shown in Fig. 5 (b), the wavelength of composition wave can

be estimated to be about 80 to 100 nm. Since this value is enough larger than the spatial resolution ($\pm$ 6 nm), it was considered that the sinusoidal compositional change is expected to be brought by the SD, not by the artifact of measurement.
